# “Guttmann Cognitest”^®^, preliminary validation of a digital solution to test cognitive performance

**DOI:** 10.3389/fnagi.2022.987891

**Published:** 2022-11-03

**Authors:** Gabriele Cattaneo, Catherine Pachón-García, Alba Roca, Vanessa Alviarez-Schulze, Eloy Opisso, Alberto García-Molina, David Bartrés-Faz, Alvaro Pascual-Leone, Josep M. Tormos-Muñoz, Javier Solana-Sánchez

**Affiliations:** ^1^Institut Guttmann, Institut Universitari de Neurorehabilitació adscrit a la UAB, Barcelona, Spain; ^2^Departament de Medicina, Universitat Autònoma de Barcelona, Bellaterra, Spain; ^3^Fundació Institut d’Investigació en Ciències de la Salut Germans Trias i Pujol, Barcelona, Spain; ^4^Departament de Medicina, Facultat de Medicina i Ciències de la Salut, Universitat de Barcelona, Barcelona, Spain; ^5^Departamento de Ciencias del Comportamiento, Escuela de Psicología, Universidad Metropolitana, Caracas, Venezuela; ^6^Hinda and Arthur Marcus Institute for Aging Research, Hebrew SeniorLife, Boston, MA, United States; ^7^Deanna and Sidney Wolk Center for Memory Health, Hebrew SeniorLife, Boston, MA, United States; ^8^Department of Neurology, Harvard Medical School, Boston, MA, United States

**Keywords:** aging, cognitive decline, cognitive functioning, computerized cognitive assessment, memory

## Abstract

Thanks to technological advances, the administration of cognitive assessments *via* digital solutions continues to increase, both in research and clinical practice. “Guttmann Cognitest”^°ledR^ is a digital solution for cognitive assessment which includes seven computerized tasks designed to assess main cognitive functions requiring approximately 20 min to be completed. The purpose of the present study was to validate it against standard and more extensive in-person neuropsychological assessments in the context of the Barcelona Brain Health Initiative (BBHI) cohort study. We studied 274 participants of the BBHI (126 women, mean age = 56.14, age range 44–69), who underwent an extensive in-person assessment, including a classical paper-and-pencil neuropsychological assessment and a cognitive assessment *via* the “Guttmann Cognitest”^°ledR^. Principal component analysis indicated that “Guttmann Cognitest”^°ledR^ measures four main cognitive domains and convergent validity analysis demonstrated that cognitive performance was associated with gold standard paper and pencil tests. Results also showed an expected negative correlation with age, a relation with educational level as well as a gender effect. Regression-based norming equations for the sample tested are also reported. Performing a cognitive assessment with this digital solution is feasible and potentially useful to gather information about cognitive functioning in large samples and experimental settings.

## Introduction

Age-related cognitive decline is one of the leading public health challenges worldwide (Minghui, 2019). In recent years, an increasing number of studies have tried to find effective strategies to prevent the development of cognitive impairment, dementia, and functional impairment due to Alzheimer’s disease and other brain-related pathologies (Amariglio et al., 2015; Cattaneo et al., 2018; Kulmala et al., 2018). A key need towards this goal is the establishment of sensitive, efficient, and accessible cognitive assessments that allow the identification of preclinical stages of diseases and the detection of subtle cognitive changes over time. Alzheimer’s disease and other neurodegenerative diseases are preceded by a long preclinical phase and the possibility to detect these cases quickly and accurately could potentially have important implications for quality of life and level of independence. For example, it will allow the implementation of preventive actions (Sternin et al., 2019) to promote resilience to pathological processes (Pascual-Leone and Bartres-Faz, 2021).

However, nowadays many of these cases remain undiagnosed, and the diagnosis of subtle symptoms in primary care is often difficult due to the lack of specially qualified personnel, resources, or optimal tools (Mortamais et al., 2017). In this scenario, mobile technologies may offer an effective solution.

Neuropsychological testing is a widely used and standardized procedure to obtain objective indicators of cognitive functioning, allowing the detection of preclinical stages of dementia (Rentz et al., 2013; Duke Han et al., 2017). Nevertheless, classical neuropsychological testing has specific limitations in terms of costs and time-consumption that make it not suitable for large-scale assessments.

The context of the COVID-19 pandemic, moreover, showed the need to find alternative ways to efficiently explore cognitive functioning, reducing people’s mobility and physical contact between clinicians and patients.

As an alternative to in-person classical neuropsychological testing, computerized assessment tools have been developed for years, and the administration of cognitive assessment *via* digital solutions continues to increase both in research and clinical practice (Tierney and Lermer, 2010; De Rover et al., 2011; Koo and Vizer, 2019), with several systems now available (O’Connell et al., 2004; Junkkila et al., 2012; Assmann et al., 2016).

This kind of assessment has been shown to offer many advantages over traditional neuropsychological testing (Zygouris and Tsolaki, 2015; Soldan et al., 2016), including saving costs and time. Moreover these tools could provide an objective and accurate recording of responses, with enhanced overall sensitivity, less dependent on professional expertise or prone to human error, and with the possibility to automatically store and compare a person’s performance between assessment sessions to, for example, trigger alerts (Dwolatzky et al., 2003; Wild et al., 2008).

However, for people not comfortable with the use of technology, computerized assessments can represent a challenge, with test interfaces appearing intimidating or counterintuitive (Zygouris and Tsolaki, 2015), making it essential to implement strategies (e.g., practice trials, clear instructions, etc.) that can reduce this bias, maximizing the possibility of a correct execution of the tests (Feenstra, 2018).

Another critical aspect is the need to validate these digital solutions in different contexts and with different populations of large enough sample sizes (Sternin et al., 2019).

This article aims to make a preliminary validation of the “Guttmann Cognitest”^°ledR^ to explore if it can represent a self-administered, useful, and efficient instrument to measure cognitive functioning in middle-aged healthy subjects for research purposes and large-scale assessments.

## Materials and Methods

### Participants

Two-hundred and seventy four participants (126 women) from the Barcelona Brain Health Initiative (BBHI, Cattaneo et al., 2018) took part in this study (see Table 1).

**Table 1:** Demographic characteristics of participants.

Variable	Mean (SD)/range	Percentage (%)
Age	56.14 (6.95)/44–69	-
Sex	-	Female: 46.0
Education level	-	Primary: 5.1
		Secondary: 27.7
		Superiors: 67.2

Participants with a history or current diagnosis of neurological or psychiatric diseases, TBI with loss of consciousness, substance abuse/dependence, treatment with psychopharmacological drugs, or visual impairments were excluded from the study. Participants provided explicit informed consent, and the protocol was approved by the Ethics and Clinical Research Committee of the Catalan Hospitals Union (Comité d’Ètica I Investigació Clínica de la Unió Catalana hospitals, CEIC18/07).

### Procedures

Participants took part in a paper-and-pencil classical neuropsychological testing session, and also a cognitive testing session *via* the digital solution “Guttmann Cognitest”^°ledR^, in the context of the in-person assessments phase of the BBHI (Cattaneo et al., 2018).

These two cognitive assessments were completed in different sessions (mean delay in days = 4.60, SD = 28.20), and their order was counterbalanced across subjects.

The “Guttmann Cognitest”^°ledR^ testing session was implemented at the Institut Guttmann facilities, partially supervised by a technician who provided the smartphone to the participant, explained the purpose of the activity, and was available for any request during the assessment. This procedure was implemented to “control” the testing setting and reduce the presence of possible bias during the validation phase.

#### Paper and pencil classical neuropsychological assessment

Two expert clinical neuropsychologists conducted the paper and pencil assessments (Vanessa Alviarez-Schulze and Alba Roca), which lasted between 60 and 90 min, with tests being administered in the same order for all participants.

The test battery included well-established neuropsychological tests, exploring main cognitive functions: general/fluid intelligence (WAIS-IV Matrix Reasoning subtest, Weschler, 2008), visuospatial searching, selective attention, visual/motor and processing speed (Cancelation test WAIS-IV, Digit symbol substitution, and Trail making test A; Reitan and Wolfson, 1985; Weschler, 2008), cognitive flexibility and set-shifting (Trail making test B; Reitan and Wolfson, 1985), working memory (Digit backward, Digit forward, Letter-number sequencing, Corsi Tap Test; Weschler, 2008), episodic memory (RAVLT; Schmidt, 1996), and visuospatial abilities (Block design; Weschler, 2008).

#### “Guttmann Cognitest”^°ledR^ digital solution assessment

The “Guttmann Cognitest”^°ledR^ is a digital solution that includes seven computerized tasks designed to assess main cognitive functions from three main domains: memory, executive functions, and visuospatial abilities.

This solution is designed to be self-administered, to be potentially used for large-scale assessments and research purposes.

After logging in, the user is presented with a welcome screen containing a short description of the testing session and its main purpose. Then, instructions to focus and pay attention on the tasks, realize the session in a quiet location, and avoid interruptions, are given. The time needed to complete the full assessment is approximately 20 min.

**Figure 1:**
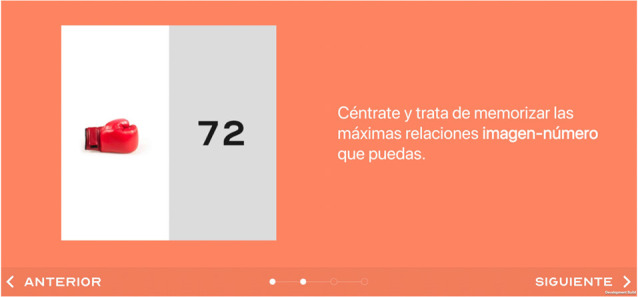
Screenshot of the video tutorial and text instructions explaining task’s logic; the user can move forward (*Siguiente*) and backwards (*Anterior*) if needed.

All tasks follow the same logic. First, an initial screen with a brief description of the task is presented. Then, more detailed instructions, together with a video tutorial, are showed, explaining the objective and rationale of the task. This tutorial can be repeated as needed and the user can also move forward and backward along it (see Figure 1). After this, the user can start the task itself.

When the user presses the start button, a countdown from 3 to 1 appears on the screen, aiming to get the user ready for the task. After this, a simple demo screen of the task comes as a practice, to ensure that the user have understood the objective, rationale, and expected responses for the task. Only once the practice is completed correctly (there are two attempts) the task begins. If the practice is not completed correctly the task is not administered, and the system moves directly to the next one, assuming that the person would not have been able to complete that task correctly. After a task is completed, a final screen displays the results obtained, and the user can continue to the next task.

Only the “Long-term memory” task (*Task 7*) does not follow this logic, as there is no tutorial nor practice screen, and the user is only informed that she/he will be required to remember the image-number associations seen in the previous “Short-term memory” task (*Task 2*).

After the seven tasks, a final questionnaire is presented to gather more information about the conditions of execution, interruptions, technical problems, or any other issues that could have affected the results of the tasks.

The seven tasks included in the solution are:

#### Task 1: visual span backward

This task, designed to assess working memory, is based on the visual span backward paradigm.

**Figure 2:**
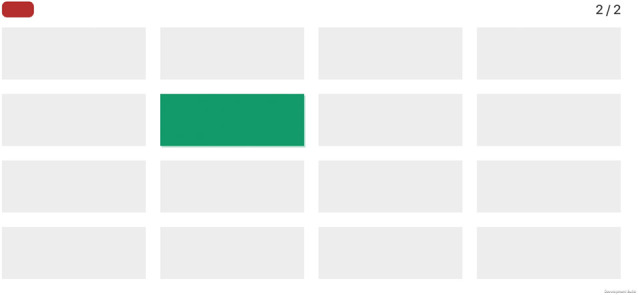
Screenshot of the visual span backward task.

Participants are instructed to memorize a sequence of lights that appears sequentially, and then repeat this sequence in reverse order. Lights appear on a grid, for a presentation time of 1 s each, and the number of elements of the series increases by one after a correct answer (Figure 2). In case of a wrong response, another sequence of the same length is presented. After two consecutive errors, the task ends, with the final score being the number of elements of the longest sequence correctly repeated.

#### Task 2: free and cued image and number associations

**Figure 3:**
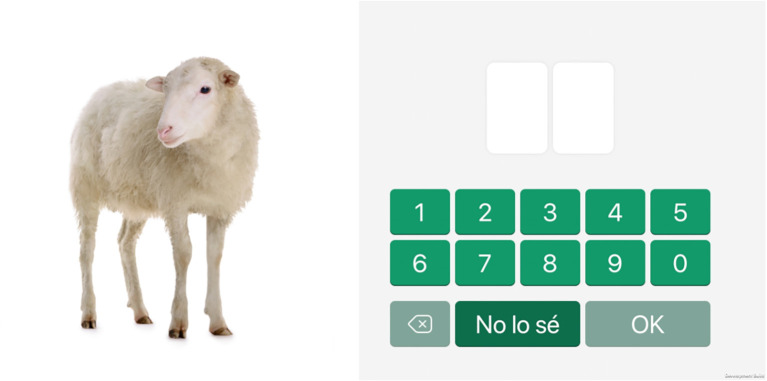
Screenshot of the free recall mode of the free and cued image and number associations task.

This associative memory task consists of memorizing six pairs of 2-digit numbers and images. The series of number-image pairs are displayed one by one, for 2 s each, in the middle of the screen. Once the presentation is finished, the images are again presented one by one, but in a different order, and participants are requested to write the number associated with each image (Figure 3).

Items that are not correctly remembered are presented again with a cue, consisting of two possible numbers associated with it, where the subject must select the correct one. The whole procedure is repeated three times.

Then, two scores are calculated for this task: one score represents the number of correct associations reported on the free recall mode, whilst the second score represents the number of correct answers on the cued mode.

#### Task 3: logic sequences

**Figure 4:**
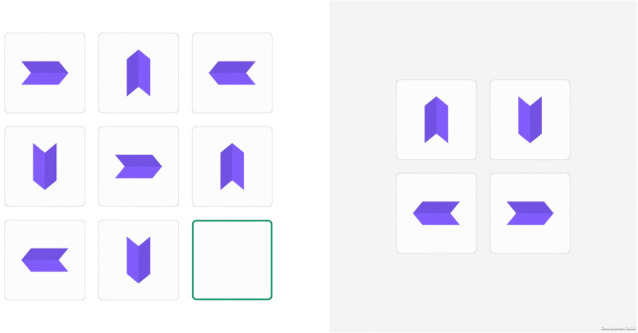
Screenshot of the logic sequences task.

Twelve logic sequences were designed to evaluate fluent intelligence and logical reasoning. Each series is composed of a 3 × 3 matrix of elements, where the element at the bottom-right is missing. The task consists of selecting one out of four possibilities, presented on the right-hand side of the screen, to complete the sequence (see Figure 4). The maximum time available to solve each series is 90 s, with an alert message appearing on the screen when there are 10 s left.

The score for this task is the number of sequences completed correctly.

#### Task 4: cancelation

The symbol cancelation test was designed to assess visuo-spatial searching and selective attention. Participants are required to select, as fast as possible, a target of a specific shape and color among a matrix of different forms. Distractors come in the form of different shapes of the same color or the same shape of a different color. The task consists of seven consecutive screens.

**Figure 5:**
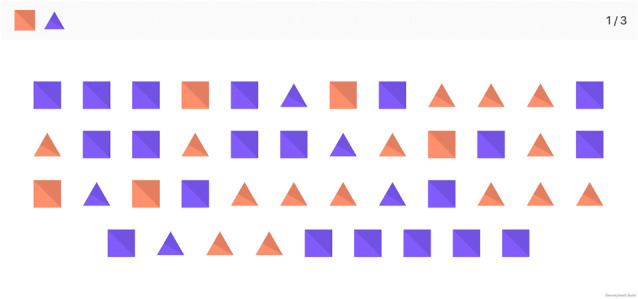
Screenshot of the cancelation task in the two targets mode.

In the first four screens, participants must select one colored shape (27 targets in total), while in the next three screens, they must choose two colored shapes (30 targets in total; see Figure 5).

There are two different ways of advancing to the next screen: either the subject selects all correct answers appearing on that screen, or the maximum 45 s threshold is reached.

The total score for this task is the sum of symbols correctly selected on all screens.

#### Task 5: circle tapping

This task was designed to measure visuomotor speed and sustained attention.

**Figure 6:**
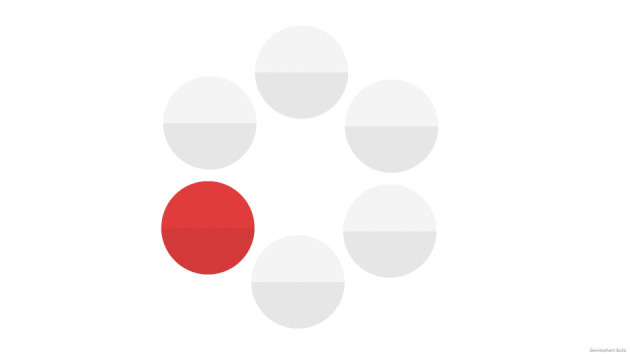
Screenshot of the circle tapping task.

This task shows six circles on the screen for 2 min. One by one, each of them appears either red or blue, with a presentation time of 1 s. The participant is instructed to only press the screen when the circles appear in red (as fast as possible; see Figure 6) and to not give any response when they appear in blue.

The calculated score for this task is determined by the total number of correct answers and the average reaction time of those correct answers in seconds.

#### Task 6: mental rotation

**Figure 7:**
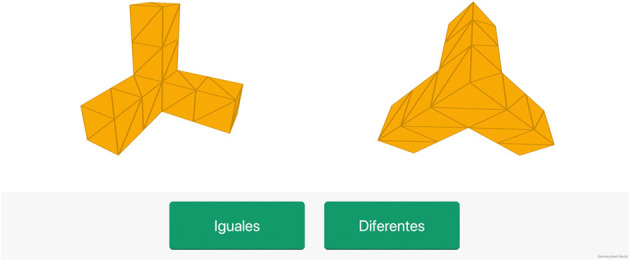
Screenshot of the mental rotation task.

This task, based on the mental rotation paradigm (Shepard and Metzler, 1971), was designed to evaluate visuospatial abilities. Participants are presented with pairs of 3D figures with different orientations, and they must indicate if they are identical or not, regardless of their orientation (see Figure 7), by pressing one of the two buttons shown at the bottom of the screen.

The task consists of 12 couple of 3D figures, and the timeout to respond is set to 10 s.

The total score of this task is the number of correct responses.

#### Task 7: long-term memory

To explore the subject’s retention capacity over time, the final task consists of presenting the same six pictures previously shown in task 2. Pictures are presented one by one, and the user must write the number associated to each picture. Similarly to the short-term task, a free recall mode comes first, whilst a cued mode is presented if the participant gives a wrong answer.

The score for this task is the number of correctly remembered associations. The mean of delay between the immediate and delayed memory tasks (Task 2 and Task 7) was 13.48 min (SD = 4.12).

### Data and statistical analysis

Following the same procedure reported in previous studies (e.g., España-Irla et al., 2021; Cattaneo et al., 2022; Redondo-Camós et al., 2022), we transformed raw scores obtained by classical gold-standard paper and pencil cognitive tests, and scores obtained in “Guttmann Cognitest”^°ledR^, into z-scores. To create composite scores of different cognitive domains, we ran two exploratory principal component analyses (one for classical cognitive testing and another one for “Guttmann Cognitest”^°ledR^) using Oblimin rotation, fixing the acceptable level of factor loading to 0.30 (Hair et al., 1998). Based on the factorial structure obtained, we calculated composite scores of the different domains as the mean of the z-scores of each neuropsychological measure, multiplying it for the component loading value, to weigh its contribution to that component. Moreover, we calculated a global cognition composite score, as the mean of all the transformed z-scores, in line with previous studies (Kaffashian et al., 2013; Lampit et al., 2015; Cattaneo et al., 2022).

Following this, we explored the relationship between socio-demographic variables and cognitive results using Spearman correlations to examine the effects of age and education, and using one-way ANOVA’s for biological sex.

Convergent validity was assessed using Spearman’s rank correlations between gold standard neuropsychological tests and results of the tasks administered with the “Guttmann Cognitest”^°ledR^ in the different cognitive domains. To explore discriminant validity, we used Steiger’s Z statistic (Hittner et al., 2003; Diedenhofen and Musch, 2015) to compare correlation coefficients.

Spearman’s rank correlations were used considering the no normal distribution of almost all cognitive scores, the fact that we did not want to make any *a priori* hypothesis about the relationship between variables (linear or monotonic), and the ordinal nature of some of the variables included in the analysis.

Furthermore, we estimated demographically adjusted based-regression norms that provided the resulting z-scores metric. Equations were obtained by calculating the predicted raw scores adjusted for sociodemographic variables (age, sex, and years of education) that resulted in the regression model that is statistically significant (Bezdicek et al., 2014; Cavaco et al., 2015; Kormas et al., 2018; Lavoie et al., 2018).

## Results

### Cognitive composite score calculation and structure

First, the results of the composite score calculation for the gold-standard paper and pencil neuropsychological assessment are presented, followed by the results obtained with the same process for the “Guttmann Cognitest”^°ledR^ digital solution assessment.

#### Paper and pencil classical neuropsychological assessment

Neuropsychological raw data of the 274 participants were transformed into z-scores and used in the principal component analysis. Bartlett’s test revealed a significant relationship between the factors (*p* < 0.001), and the Kaiser-Meyer-Olkin (KMO) test confirmed that the data was suitable for principal component analysis (KMO = 0.62). To select the number of components we used the eigenvalue-one Kaiser’s criterion (Kaiser, 1960), controlling also the cumulative variance explained.

The analysis resulted in four components that explained 60.89% of the variance (see Table 2). The first component included the digit span backward (0.75), digit span forward (0.68), letter-number sequencing tests (0.68), Corsi Tap Test (0.37), and WAIS-IV logical Matrices (0.59), indicating an executive functions domain embracing working memory and reasoning abilities.

**Table 2:** Results of formal neuropsychological testing of participants and principal components structure.

Cognitive domain	Neuropsychological test	*Mean (SD)*
Executive fcuntions	Digit span forward	6.20 (1.20)
	Digit span dackward	5.01 (1.22)
	Letter-number sequencing	5.72 (1.31)
	Corsi tap test	6.64 (1.21)
	WAIS-IV logical matrices	20.09 (3.56)
Episodic memory	RAVLT immediate recall	55.78 (9.34)
	RAVLT delayed recall	12.30 (2.56)
	RAVLT recognizing	14.55 (1.01)
Visuo spatial searching/attention	WAIS-IV block design	46.72 (10.25)
	Digit symbol substitution	77.20 (13.84)
	WAIS-IV cancelation	41.78 (8.82)
	TMT A	26.23 (7.70)
Set shifting	TMT B	79.44 (28.76)
	TMT B-A	53.20 (26.19)

A second episodic memory domain was composed of all the measures of the Rey Auditory Verbal Learning Test (immediate recall = 0.83, delayed recall = 0.90, and recognition = 0.77).

A third visuospatial searching and attentional component comprised of the Trail Making Test A (0.72), the block design test (0.58), the digit-symbol substitution test (0.78), and the cancelation test (0.69).

Finally, set-shifting abilities were reflected in a fourth component, with the Trail Making Test Part B (0.81) and the Trail Making Test Part B-A (0.87). As mentioned above, based on this factorial structure we calculated composite scores of the four domains, and a global cognition score, as the mean of all tests.

#### “Guttmann Cognitest”^°ledR^ digital solution assessment

Bartlett’s test revealed a significant relationship between the factors (*p* < 0.001), and the Kaiser-Meyer-Olkin (KMO) test confirmed that the data was acceptable for principal component analysis (KMO = 0.59). As in the previous analysis, the number of components was selected using Kaiser’s rule (Kaiser, 1960), checking for the cumulative variance explained.

The analysis resulted in four components that explained 70.27% of the variance (see Table 3). A first domain was composed by logic sequences (0.71), cancelation (0.67), and visual span backward (0.65) tasks, possibly reflecting attention and executive functions (attention, reasoning, and working memory).

**Table 3:** Principal components structure for the Cognitest app.

Cognitive domain	Cognitest task
Executive fcuntions and attention	Logic sequences
	Visual span backward
	Cancelation
Episodic memory	Free recall
	Cued recall
	Delayed recall
Visuomotor speed	Circle tapping accuracy
	Circle tapping reaction time
Visuospatial processing	Mental rotation

The second component included free recall (0.86), cued recall (0.84), and delayed recall (0.75) of images and numbers association tasks, indicating a potential memory component.

A third visuomotor speed component included the circle tapping accuracy (0.92) and reaction times (0.93).

The last component comprised only the mental rotation task (0.87).

Composite scores were calculated as the mean of the z-scores of each task, and a global score as the mean of all tasks results.

### Age, sex, education, and cognition

Age correlated negatively with results of the “Guttmann Cognitest”^°ledR^ digital solution in memory (*rs* = −0.20, *p* < 0.001), executive functions and attention (*rs* = −0.35, *p* < 0.001), visuomotor speed (*rs* = −0.30, *p* < 0.001), and mental rotation (*rs* = −0.14, *p* = 0.027). Also, the global score in the “Guttmann Cognitest”^°ledR^ was negatively associated with age (*rs* = −0.48, *p* < 0.001).

We found a positive correlation between education and results obtained in memory (*rs* = 0.28, *p* < 0.001), executive functions and attention (*rs* = −0.15, *p* = 0.009), and the global cognitive score (*rs* = 0.25, *p* < 0.001).

Regarding biological sex differences, males outperformed women in executive functions and attention (0.19 and −0.22 respectively; *F* = 8.457, *p* = 0.001, η^2^ = 0.032), visuomotor speed (0.14 and −0.17; *F* = 3.883, *p* = 0.050, η^2^ = 0.015), mental rotation (0.17 and −0.20; *F* = 8.041, *p* = 0.005, η^2^ = 0.031) and global cognition (0.11 and −0.12; *F* = 8.496, *p* = 0.004, η^2^ = 0.033). On the other hand, women outperformed males in memory performance (0.98 and −0.08 respectively; *F* = 4.150, *p* = 0.043, η^2^ = 0.016).

For gold-standard and classical neuropsychological tests, age correlated negatively with visuospatial searching and attention (*rs* = −0.43, *p* < 0.001), executive functions (*rs* = −0.28, *p* < 0.001), and global cognition (*rs* = −0.29, *p* < 0.001).

Regard education we found positive correlations with results in memory (*rs* = 0.22, *p* < 0.001), visuo-motor speed (*rs* = 0.23, *p* < 0.001), executive functions (*rs* = 0.23, *p* < 0.001), and global cognition (*rs* = 0.30, *p* < 0.001).

When we explored sex differences, similarly with what found previously, women outperformed males in memory (0.18 and −0.15 respectively; *F* = 8.805, *p* = 0.003, η^2^ = 0.032), whereas men performed better than women in executive functions (0.28 and −0.33 respectively; *F* = 26.787, *p* < 0.001, η^2^ = 0.091), set shifting (0.12 and −0.15 respectively; *F* = 5.801, *p* = 0.017, η^2^ = 0.021), and global cognition (0.07 and −0.09 respectively; *F* = 4.909, *p* = 0.028, η^2^ = 0.018).

### Convergent validity

To explore convergent validity, cognitive domains calculated with the results of the “Guttmann Cognitest”^°ledR^ digital solution were correlated with those obtained by paper and pencil tests (see Table 4).

**Table 4:** Correlations between results of the “Guttmann Cognitest”^°ledR^ digital solution and those obtained by classical paper and pencil neuropsychological testing.

**Gold-standard paper and pencil tests**							
		*Memory*	*Executive functions*	*Visuo-spatial searching and attention*	*Set-shifting*	*Global cognition*
**“Guttmann Cognitest”^°ledR^**	*Memory*	*rs* = 0.27**	*rs* = 0.11**	*rs* = 0.18**	*rs* = 0.14**	*rs* = 0.19**
	*Executive functions and attention*	*rs* = 0.13**	*rs* = 0.37**	*rs* = 0.24**	*rs* = 0.03**	*rs* = 0.37**
	*Visuo-motor speed*	*rs* = 0.04**	*rs* = 0.09**	*rs* = 0.26**	**rs = −0.22**	*rs* = 0.06**
	*Mental rotation*	*rs* = −0.07	*rs* = 0.10**	*rs* = 0.17**	*rs* = 0.03**	*rs* = 0.09**
	*Global cognition*	*rs* = 0.19**	*rs* = 0.37**	*rs* = 0.36**	*rs* = 0.14**	*rs* = 0.36**

Correlation coefficients in bold represents higher correlations, at a statistically significant level. *p < 0.05, **p < 0.001.

### Memory measured by “Guttmann Cognitest”^°ledR^

Memory correlated with results of the gold-standard paper and pencil assessment in memory (*rs* = 0.27, *p* < 0.001), visuospatial searching and attention (*rs* = 0.18, *p* = 0.004), set-shifting (*rs* = 0.14, *p* = 0.021), and global cognition (*rs* = 0.19, *p* = 0.002).

When we compared correlation magnitudes (Hittner et al., 2003), we found that the correlation between memory measured by “Guttmann Cognitest”^°ledR^ and the same domain measured by classical neuropsychological tests was higher compared with the correlation between memory measured by “Guttmann Cognitest”^°ledR^ and set-shifting (*z* = 1.68, *p* = 0.046). However, it was similar in magnitude compared to the correlation with visuo-spatial searching and attention, and global cognition measured with gold-standard tests.

### Attention and executive functions measured by “Guttmann Cognitest”^°ledR^

Attention and executive functions measured by “Guttmann Cognitest”^°ledR^ correlated with results of gold-standard paper and pencil neuropsychological tests in episodic memory (*rs* = 0.13, *p* = 0.034), visuospatial searching and attention (*rs* = 0.24, *p* < 0.001), executive functions (*rs* = 0.37, *p* < 0.001), and global cognition (*rs* = 0.37, *p* < 0.001). When we compared correlations magnitude (Hittner et al., 2003) we found that the correlation between executive functions measured by “Guttmann Cognitest”^°ledR^ and the same domain measured by classical gold-standard measures was greater than the correlation between executive functions measured by “Guttmann Cognitest”^°ledR^ and memory (*z* = 2.91, *p* = 0.002), and visuo-spatial searching (*z* = 1.78, *p* = 0.038). However, it produced similar results in terms of magnitude in relation to the correlation with global cognition.

### Visuomotor speed measured by “Guttmann Cognitest”^°ledR^

This component correlated with classical tests of visuospatial searching and attention (*rs* = 0.25, *p* < 0.001) and set-shifting abilities (*rs* = 0.22, *p* < 0.001), with similar magnitude.

### Mental rotation measured by “Guttmann Cognitest”^°ledR^

Mental rotation results correlated only with executive functions measured with classical neuropsychological tests (*rs* = 0.17, *p* = 0.008).

### Global cognition measured by “Guttmann Cognitest”^°ledR^

The Global “Cognitest” composite score correlated with gold-standard tests of episodic memory (*rs* = 0.19, *p* = 0.003), visuo-spatial searching and attention (*rs* = 0.36, *p* < 0.001), executive functions (*rs* = 0.37, *p* < 0.001), set shifting (*rs* = 0.14, *p* = 0.025), and global cognition (*rs* = 0.36, *p* < 0.001). In relation to the magnitude of the correlation between global composite scores, it resulted in higher than the correlation between the Global “Cognitest” composite score and classical tests of memory (*z* = 2.84, *p* = 0.002) and set-shifting (*z* = 3.33, *p* < 0.001).

## Regression-based norming equations

We estimated regression-based norms that provided z-score metrics for each of the tasks included in the “Guttmann Cognitest”^°ledR^ digital solution. Equations included sociodemographic variables that were statistically significant in the regression model (for biological sex we created a dummy variable; see Table 5 for values used), multiplied for not standardized “B” coefficients, model constant (k), and the Root Mean Square Error (RMSE):


$$z=\frac{Raw\;Score-[k+(B_{age}*Age)+(B_{sex}*sex)+(B_{education}*Education]}{RMSE}$$

**Table 5:** Variables included in the equations and values to be used in the regression-based norms formulae.

Variables		Values
Age		age in years
Sex	Male	0
	Female	1
Education level	Primary (≤8 years)	1
	Secondary (9–12 years)	2
	Superiors (≥13 years)	3

### Task 1: visual span backward


$$z=\frac{Raw\;Score-[7.623+(-0.060*Age)+(-0.404*sex)]}{1.361}$$

### Task 2: free and cued images and numbers associations

Free recall


$$z=\frac{Raw\;Score-[12.904+(-0.136*Age)+(1.076*sex)+(1.398*Education]}{3.164}$$

Cued recall


$$z\text{ = }\frac{Raw\;Score-[16.573+(-0.035*Age)+(0.628*sex)+(0.587*Education)]}{1.707}$$

### Task 3: logic sequences


$$z=\frac{Raw\;Score-[12.020+(-0.103*Age)+(-1.010*sex)+(1.009*Education)]}{2.642}$$

### Task 4: cancelation


$$z=\frac{Raw\;Score-[67.884+(-0.246*Age)+(-0.163*sex)+(-1.240*Education)]}{11.604}$$

### Task 5: circle tapping

Accuracy (%)


$$z=\frac{Raw\;Score-[93.934+(-0.042*Age)]}{1.288}$$

Reaction time (seconds)


$$z=\frac{Raw\;Score-[0.343+(-0.006*Age)+(0.032*sex)]}{0.099}$$

### Task 6: mental rotation


$$z=\frac{Raw\;Score-[12.062+(-0.782*sex)]}{2.457}$$

### Task 7: long-term memory


$$z=\frac{Raw\;Score-[5.934+(-0.018*Age)+(0.237*Education)]}{0.680}$$

## Discussion

This study aimed to make a preliminary validation of the “Guttmann Cognitest”^°ledR^ digital solution as a tool for measuring cognitive functioning in a sample of healthy middle-aged adults. Results showed that it is a useful and suitable instrument for this population.

Principal component analysis on “Guttmann Cognitest”^°ledR^ tasks showed the presence of four main components: memory, executive functions and attention, visuomotor speed, and mental rotation. Moreover, it was possible to calculate a global score reflecting unspecific global cognitive functioning.

Similarly, also for classical neuropsychological tests, the analysis showed the presence of four main components, corresponding to the cognitive domains of memory, executive functions, visuospatial searching and attention, and set-shifting.

Beyond similarities, the components obtained by the two analyses did not include the same exact test, and consequently did not completely overlap in terms of cognitive processes involved.

For example, the domain of executive function for the classical neuropsychological tests includes principally working memory tests, together with a reasoning test, while the “correspondent” component in the “Guttmann Cognitest”^°ledR^ includes a working memory task, a reasoning task, but also a cancelation task.

A possible reason for this could be the greater involvement of working memory, and/or inhibition processing, in the cancelation task we designed. Indeed it has been proposed that working memory could be involved in visual selective attention (De Fockert et al., 2001) and strongly related to inhibition (McNab et al., 2008).

However, even considering these potential differences in terms of sub-processes involved in each task, we consider that, in terms of “broad” cognitive domains and functions, the overlapping between cognitive domains measured with “Guttmann Cognitest”^°ledR^ and classical neuropsychological tests should be considerable.

Crucially, convergent validity analysis indicated that these domains were associated with the correspondent and expected domains measured by gold-standard paper and pencil neuropsychological tests.

In order to interpret these correspondences we specifically considered the magnitude of the effect sizes obtained in the correlation analyses, following the criteria proposed by Cohen (1988); see also Hemphill (2003).

For memory tasks, we found small to medium correlations with all cognitive domains, except executive functions, with the strongest of these being episodic memory, visuo-spatial searching and attention, and global cognition.

Another component measured by “Guttmann Cognitest”^°ledR^, reflecting attention and executive functions, showed medium correlations with classical tasks measuring similar attentional and executive components and the global cognition composite score, while showed only small correlations with visuo-spatial searching and memory classically measured.

The visuomotor speed component correlated with tests related to visuospatial searching and attention and set-shifting with coefficients small in magnitude.

Finally, the mental rotation component only showed a small correlation with the executive function component. This could be explained considering that this test measures mainly visual ability and visual imagery (Campos, 2012) that were not assessed in the neuropsychological testing, with only a low engagement of executive functions (Hyun and Luck, 2007). Future studies must include these tests to evaluate the convergent validity of the mental rotation task.

These results are in line with previous validation studies of computerized neuropsychological testing (see Gualtieri and Johnson, 2006; Tsoy et al., 2021 for reviews) , showing great variability in convergent validity and concurrent validity, varying from small to large effect sizes (from 0.2 to 0.88 with an average of 0.40).

This variability makes it somehow difficult to make clear *a priori* expectation about desirable effect sizes for a useful solution, but from our point of view coefficients lower than 0.2, even if statistically significant, should be interpreted with caution.

Considering all the obtained results, beyond differences in magnitude between the different components, the “Guttmann Cognitest”^°ledR^ showed a satisfactory overall convergent validity with gold-standard correspondent neuropsychological tests.

Divergent validity analysis showed a certain overlap between components, and almost all cognitive domains measured by “Guttmann Cognitest”^°ledR^ correlated with visuospatial searching and attention measured by gold-standard paper and pencil tests. This finding indicates that this cognitive component represents a subjacent and common cognitive process involved in all tasks, in line with the involvement of specific cognitive processes (e.g., working memory) in tasks designed to mainly measure other cognitive functions (e.g., visual searching and attention; De Fockert et al., 2001).

However, the same patterns of results were found for classical paper and pencil tests, and the relationship and overlap between different cognitive processes involved in different tests is a widely debated issue, exhaustively explored in the past (Vanderploeg et al., 1994; Cunningham et al., 1997; Fossati et al., 1999; Tremont et al., 2000; Bryson et al., 2001).

In clinical populations, for example, a clear dissociation between deficits in different cognitive domains is not always detectable, a fact that can be explained due to different levels of overlapping depending on the tests used (Fossati et al., 1999; Tremont et al., 2000).

Trying to quantify this overlap, Duff et al. (2005) found a strong relationship between different cognitive domains that explained up to 59% of the shared variance.

For these reasons, it is sometimes appropriate to calculate a global cognitive score that could be fully informative of general cognitive functioning, and therefore, provide valuable information for population screening or in large sample studies.

Regarding the relation with socio-demographic variables, we found that age, as expected, correlated negatively with performance in all cognitive domains, indicating a sort of sensitivity of these tasks to cognitive changes due to age. On the other hand, education correlated positively with memory, executive functions, and global cognition.

Interestingly, males outperformed women in all cognitive domains apart from memory, where women performed better. One possible interpretation of these results could be related to the great involvement of visuospatial components in all the task of the “Guttmann Cognitest”^°ledR^ digital solution, but similar results were observed also for the classical neuropsychological tests. However, in both cases, the effect sizes were very small, and, in line with what was reported above, the observed differences must be interpreted with caution.

This is particularly important if we consider reviews and meta-analysis on this topic showing that beyond a consistently observed advantage for males in mental rotation (Hyde, 2014), differences in other cognitive domains are often very heterogeneous (Gaillard et al., 2021), trivial in effect size (Hyde, 2014), and not supported by differences in brain anatomy or brain functioning (Jäncke, 2018).

To conclude, our results showed that the “Guttmann Cognitest”^°ledR^ digital solution is a suitable instrument to measure cognitive functioning in different domains for research purposes and large samples assessments of middle-aged adults, supporting the potential relevance of these kinds of tools for large-scale assessment.

Digital solutions like this one indeed could serve as a reference point for further, more specialized, testing. Despite their limitations, they could provide a brief picture of cognitive functioning, helping to detect early impairments in specific domains in large-scale samples. In this context, these “screening” tests should focus on functions that deteriorate first in the preclinical stage of cognitive disorders (Payton et al., 2020). For this reason, we decided to include very sensitive tests previously related to the early detection of Alzheimer’s Disease (AD), like the associative memory task, sensitive to the medial temporal lobe dysfunctions (O’Connell et al., 2004; De Rover et al., 2011; Junkkila et al., 2012; Soldan et al., 2016).

However, this study presents several limitations that should be considered. First, due to the difference between the set of tasks included in our digital solution and the classical neuropsychological tests used to validate it (e.g., associative memory task vs. word list), the cognitive component calculated may not totally overlap and reflect exactly the same subjacent cognitive processes. Second, even if we try to weigh the contribution to each test to a cognitive domain, each task could contribute differently to a single domain and the different domains could contribute differently to the global cognitive score calculated. Third, the neuropsychological tests included to validate “Guttmann Cognitest”^°ledR^ tasks did not include tests that mainly measure visuospatial abilities and visual imagery, making difficult a proper validation of the mental rotation task.

Further work is needed to examine the use of this kind of cognitive assessment in clinical populations of older adults, alongside the validation of the test-retest reliability and learning effect of these tests. In this regard, two versions of each task with different stimuli have already been designed, and a specific validation study is already underway. We view this as a crucial aspect to allow us to efficiently implement cognitive assessments and follow-ups over time.

## Data Availability Statement

The raw data supporting the conclusions of this article will be made available by the authors, without undue reservation.

## Ethics Statement

The studies involving human participants were reviewed and approved by Ethics and Clinical Research Committee of the Catalan Hospitals Union—Comité d’Ètica I Investigació Clínica de la Unió Catalana hospitals, CEIC18/07. The patients/participants provided their written informed consent to participate in this study.

## Author Contributions

GC, JS-S, AG-M, EO, and JT-M participated in the initial conception of the design of the “Guttmann Cognitest”^°ledR^ digital solution. GC, JS-S, VA-S, CP-G, and AR participated actively in the data collection and analysis. AG-M, EO, DB-F, AP-L, and JT-M contributed to the interpretation of the results. GC and CP-G drafted the article and all other authors made critical revisions, introducing important intellectual content. All authors contributed to the article and approved the submitted version.

## Funding

DB-F was funded by the Spanish Ministry of Science, Innovation, Universities (RTI2018-095181-B-C21) and ICREA Academia 2019 award research grants. JT-M was partly supported by Fundació Joan Ribas Araquistain_Fjra, AGAUR, Agència de Gestió d’Ajuts Universitaris i de Recerca (2018 PROD 00172), Fundació La Marató De TV3 (201735.10), and the European Commission (Call H2020-SC1-2016-2017_RIA_777107).

## Conflict of Interest

The authors declare that the research was conducted in the absence of any commercial or financial relationships that could be construed as a potential conflict of interest.

## Publisher’s Note

All claims expressed in this article are solely those of the authors and do not necessarily represent those of their affiliated organizations, or those of the publisher, the editors and the reviewers. Any product that may be evaluated in this article, or claim that may be made by its manufacturer, is not guaranteed or endorsed by the publisher.
